# Love Laughs at Laws in Wisconsin

**Published:** 1914-02

**Authors:** 


### EDITORIAL DEPARTMENT.

DR. F. E. DANIEL, Editor DR. R. H. L. BIBB, Associate Editor

DEPARTMENTS { EUGENICS: Drs. M. Duggan and T. Y. Hull.

WOMAN'S DEPARTMENT: Mrs. F. E. Daniel.

LOVE LAUGHS AT LAWS IN WISCONSIN.—The framers of the Wisconsin marriage law so far overshot the mark as to defeat the purpose for which it was intended, and really make it ridiculous. It has put a stop to marrying in Wisconsin; that is, by the license plan of marriage, and those who desire to be married by priest or parson, have simply to cross the line into another State. No one, it seems, will submit to the examination prescribed by the law—a laboratory examination of blood tests and reactions—and no doctor seems willing to make such examination for $3.00, the fee prescribed by the law. The law is thus a dead letter, and will have to be repealed. It has had one bad effect; it caused the Legislature of South Carolina to defeat a really rational bill, and no doubt other States will be influenced by this *faux pas,* and the subject of marriage regulation will receive a backset.

But, Wisconsin lovers laugh at the law, and evade it, as shown above, and there are those, and perhaps the majority, who say: "Why have a license?"; "why have a preacher?" since the State recognizes common law marriages, and the courts have upheld them. Two persons have only to agree to live together as man and wife, make a written contract and sign it in the presence of witnesses, and they are as much married as if the "knot" had been tied by a dozen priests, bishops, or prelates; they require no license from the State, the clerk of the court records the contract, charges a fee of 10 cents, and the thing is done; no license; no examination!

The essence of marriage is only consent, and this evidence and record of consent is just as sacred and as binding as if it had the religious sanction. That is only a fashion, and church weddings are the custom. In slave-days the couple had only to ask "Marster's" consent, and the only ceremony was jumping over a broomstick or something similar.

While Wisconsin has been too radical, Texas has been too conservative, and every effort on the part of the Texas doctors to induce the Legislature to enact a much-needed marriage law, has been a failure. At the mention of it, some of them get cold feet, and back off. It is a difficult matter to get them to listen to such propositions. Or, if a bill gets before a committee, and is reported favorably, some fellow will kill it in the House or Senate by misrepresentation, or a lot of sentimental gush, as they did the sterilization bill last Legislature. It had passed the House, was up in the Senate, when a fellow, who had not even read the bill, killed it by reading into it something foreign to its object and purpose. He said it was "cruel and unusual punishment," which is "unconstitutional." The word "punishment" does not occur in the bill. By the bye, the courts of New Jersey have decided against the excellent sterilization bill passed by their last Legislature; pronounced it "unconstitutional."

